# *Austromesocypris bluffensis* sp. n. (Crustacea, Ostracoda, Cypridoidea, Scottiinae) from subterranean aquatic habitats in Tasmania, with a key to world species of the subfamily

**DOI:** 10.3897/zookeys.215.2987

**Published:** 2012-08-17

**Authors:** Ivana Karanovic, Stefan Eberhard, Giulia Perina

**Affiliations:** 1 Hanyang University, Department of Life Science, Seoul 133-791, Korea and Institute of Marine and Antarctic Studies, University of Tasmania, Private Bag 49, Hobart, Tasmania 7001, Australia; 2 Subterranean Ecology, Scientific Environmental Services, Suite 8, 37 Cedric Street, Stirling, Western Australia 6021, Australia

**Keywords:** Ostracods, biodiversity, Cyprididae, Australia

## Abstract

*Austromesocypris bluffensis* **sp. n.** is described and we report another species, *Austromesocypris* sp., both collected from subterranean aquatic habitats in Tasmania. This discovery adds a major taxonomic group to the already diverse invertebrate cave fauna of Tasmania, and is of interest because, globally, obligate subterranean aquatic species (stygobites) are poorly represented within the family Cyprididae. The genus *Austromesocypris* Martens, De Deckker & Rossetti, 2004 is otherwise known to comprise entirely “terrestrial or semi-terrestrial” species. The second species is not described because only juvenile specimens were collected. Both species stand apart from their congeners by the carapace shape, which is rectangular in *Austromesocypris bluffensis* and triangular and asymmetrical in the unnamed species. Another unique feature of the new species is the almost symmetrical uropodal rami. We also identify some broader systematic issues within the Scottiinae including the position of two New Zealand species, *Scottia audax* (Chapman, 1961) and *Scottia insularis* Chapman, 1963 in the genus, and point out their closer relationship to the Gondwana genera of Scottiinae, *Austromesocypris* and *Mesocypris* Daday, 1910, than to the Palearctic genus *Scottia* Brady & Norman, 1889, based on the morphology of the maxillula and mandibula. The identity of the Australian records of *Scottia audax* (Chapman, 1961), *Austromesocypris australiensis* (De Deckker, 1983) and the Boreal records of *Scottia pseudobrowniana* Kempf, 1971 are all considered doubtful. A key to the world species of Scottiinae is provided.

## Introduction

Twelve freshwater podocopid ostracods belonging to the superfamily Cypridoidea and family Cyprididae are known from Tasmania (Table 1). The family Candonidae and representatives of the superfamily Cytheroidea, chiefly the family Limnocytheridae, have been recorded from Quaternary deposits (De Deckker 1982b) from Pulbeena and Mowbray swamps in north-west Tasmania. Darwinulidae are represented in Tasmania by one unnamed species of *Penthesilenula* Rossetti and Martens, 1998 (see Martens and Rossetti 2002) from the same Quaternary site.

**Table 1.** List of ostracod species recorded from Tasmania. R - Recent, F - Fossil.

**Species**	**Record**	**Reference**
Superfamily Cytheroidea		
*Australocypris robusta* (De Deckker, 1974)	R	De Deckker (1977)
*Candonocypris incosta* De Deckker, 1982	R	De Deckker (1982)
*Diacypris spinosa* De Deckker, 1981	R	De Deckker (1981)
*Diacypris dietzi* (Herbst, 1958)	R	Herbst (1958)
*Kennethia cristata* De Deckker, 1979	R	De Deckker, 1979
*Mytilocypris praenuncia* (Chapman, 1966)	R	Halse and McRae (2004)
*Mytilocypris splendida* (Chapman, 1966)	R	De Deckker (1977)
*Mytilocypris tasmanica* McKenzie, 1966	R	McKenzie (1966), De Deckker (1977), Halse and McRae (2004)
*Mytilocypris mytiloides* (Brady, 1886)	R	De Deckker (1977)
*Mesocypris tasmaniensis* De Deckker, 1983	R	De Deckker (1983)
*Newnhamia fenestrata* King, 1855	R	De Deckker (1978)
*Sarscypridopsis aculeata* (Costa, 1847)	R	De Deckker (1982a)
*Candona tecta* De Deckker, 1982	F	De Deckker (1982b)
*Candonopsis tenuis* (Brady, 1886)	F	De Deckker (1982b)
Superfamily Cytheroidea		
*Limnocythere mowbrayensis* Chapman, 1914	F	De Deckker (1982b)
*Gomphodella australiaca* (Hussainy, 1969)	F	De Deckker (1982b)

Since their first appearance in the early Palaeozoic, podocopid ostracods have occurred in both marine and freshwater habitats, and today can be found from abyssal depths to small freshwater puddles, and even terrestrial environments (Pinto et al. 2003, 2004, 2005, 2008; Martens et al. 2004). All three major podocopid lineages, Cypridocopina, Darwinulocopina and Cytherocopina, have several representatives living in terrestrial habitats. Although Darwinulidae has no species restricted exclusively to terrestrial habitats, several species of the genera *Vestalenula* Rossetti and Martens, 1998 and *Penthesilenula* Rossetti and Martens, 1998 have been collected from terrestrial environments in Brazil (Pinto et al. 2003, 2004, 2005) (for the review of “semi-terrestrial ostracods” see Appendix).

Schornikov (1980) argued that ostracods found in terrestrial environments cannot be considered true terrestrial animals, as they were always covered by water, even if only a drop. This claim was rejected by Pinto et al. (2005) and Martens et al. (2004), who considered “terrestrial” all animals living outside free standing or flowing water. Danielopol and Vespremenau (1964) observed *Scottia pseudobrowniana* (Jones, 1850) move towards more humid areas, but closed its valves and stopped moving in non-saturated conditions, only becoming active again when in contact with water. Individuals of *Scottia audax* (Chapman, 1961)tended to move towards more damp parts of the litter as the available water decreased (Chapman 1961). Horne et al. (2004) recorded strong hydrotaxis in *Terrestricythere elisabethae* Horne, Smith, Whittaker and Murray, 2004, and death if desiccated for more than 10 minutes. The ability to inhabit environments such as leaf litter and other damp habitats may be advantageous, since it can allow a species to widen its area of distribution significantly and avoid predators occurring in fully aquatic habitats. It is easy to imagine how these tiny animals move, and one direct example is the finding of *Microdarwinula zimmeri* (Menzel, 1916) and *Scottia pseudobrowniana* on the floating fen soil (always in the part of the fen covered with water) in Romania (Danielopol and Vespremenau 1964). On the other hand, this way of life has its obvious limits, since such places are often subject to rapid desiccation. One more argument for not considering these ostracods truly adapted to the terrestrial environment is the fact that they are actually not widely distributed as would be expected with this adaptation. We know of only three exceptions where significantly broader distribution ranges, including trans-continental, are implied: *Mesocypris pubescens* Daday, 1910 has been recorded from Mount Kilimanjaro (Daday 1910), Kenya (Klie 1939) and South Africa (Lawrence 1953); *Scottia audax* from New Zealand (Chapman 1961) and widely throughout eastern Australia (De Deckker 1980, 1983); and *Scottia pseudobrowniana* from Europe, Asia (see Meisch 2000) and North America (Cole 1966, Külköylüoglu and Vinyard 2000). The broad distribution of these three species is discussed further in this paper and it is shown that they actually comprise additional unrecognized species. Redescriptions of these species is beyond the scope of this paper, but “species” as recognized herein are included in the key to species of Scottiinae presented at the end of this paper.

Animals living away from free-standing or flowing water have usually very hirsute shell and soft parts, which probably allows them to retain enough moisture in semi-terrestrial surroundings (Horne et al. 2004). Pinto et al. (2005) also consider parthenogenetic reproduction, commonly found in these ostracods, as one of the pre-adaptations to this environment. Although Chapman (1961) did not provide a detailed taxonomic description of *Mytilocypris audax*, she gave a very interesting observation on the behaviour of these animals. She suspected that the juveniles hatch inside mother's shell, since she observed large eggs within the shell with the outline of valves showing. Even more, in a live culture, in which young specimens were suddenly noticed, no laid eggs were seen. All this may suggest brooding care, never noticed before in Cypridoidea.

During the recent study of Tasmanian caves, two interesting new species were collected. One is named and described here, while the other is only briefly described but left as an open nomenclature, because only a juvenile specimen was collected. They both belong to the genus *Austromesocypris* Martens, De Deckker and Rossetti, 2004, a very peculiar and otherwise entirely “terrestrial” genus known only from Australia (Martens et al. 2004).

## Study area

**Figure 1.**
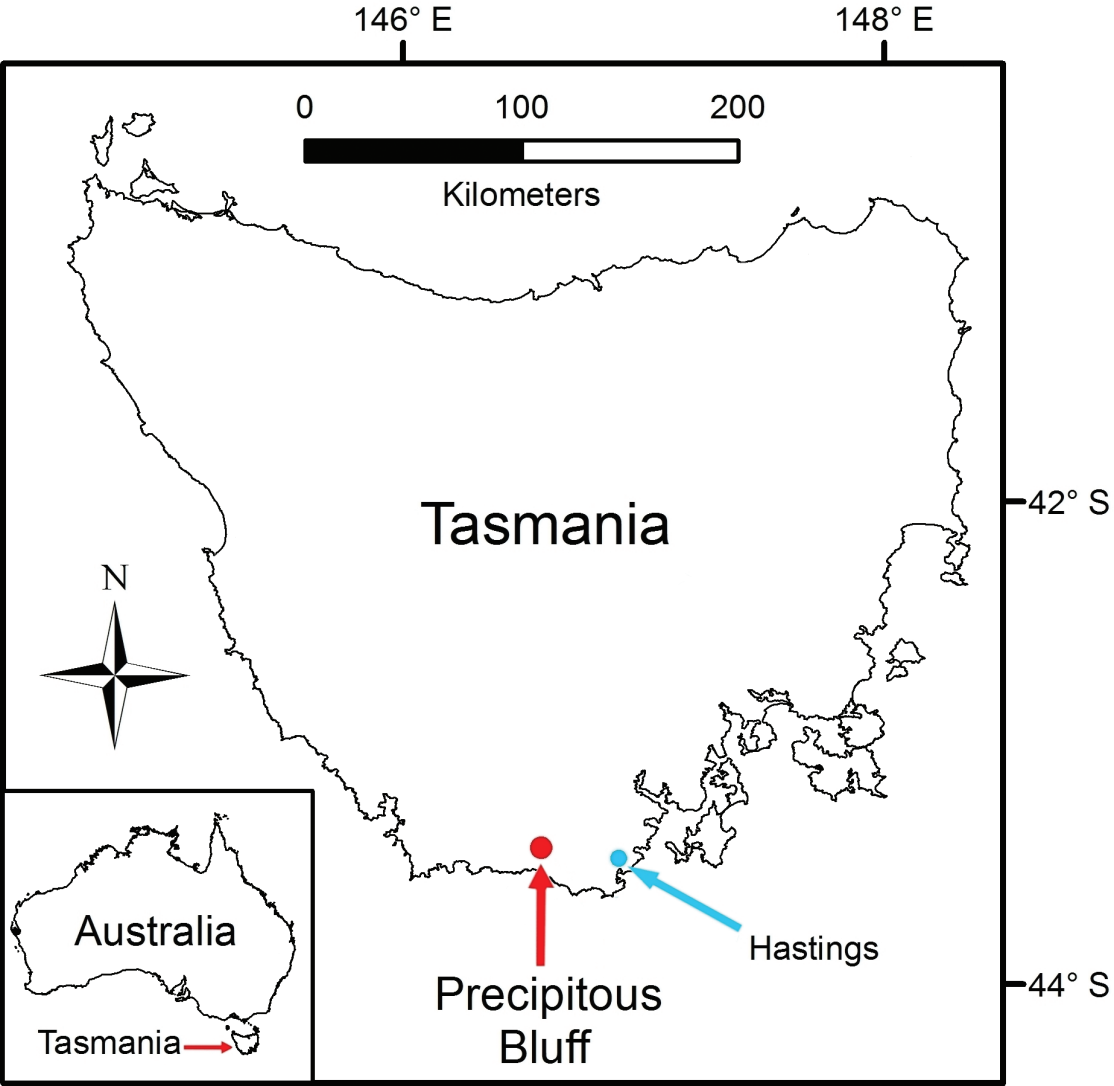
Tasmania, Australia, showing location of Precipitous Bluff study area.

**Figure 2.**
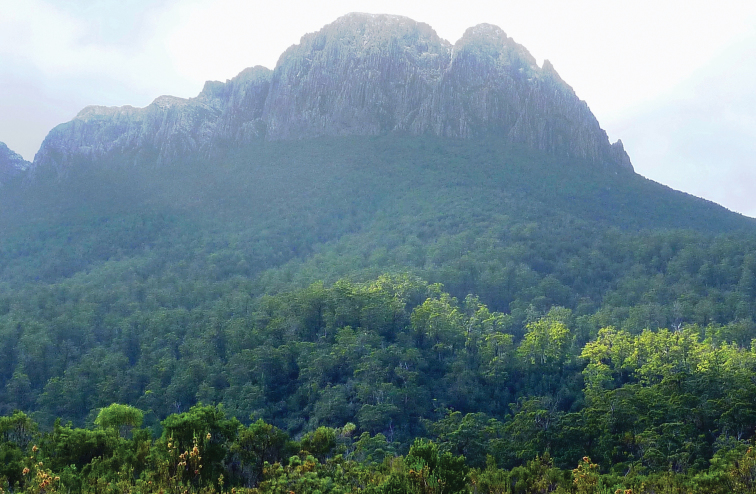
Precipitous Bluff (1140 m asl) showing heavily forested lower slopes which contain the karst and caves.

**Figure 3.**
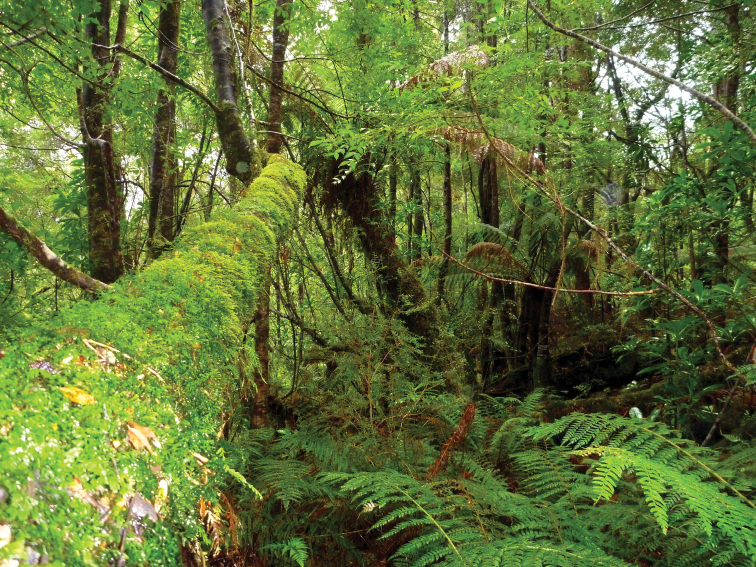
Typical cool temperate rainforest overlying the karst and caves.

Precipitous Bluff is located near the south coast of Tasmania in a remote and inaccessible part of the Tasmanian Wilderness World Heritage Area (Figure 1). The area remains essentially undisturbed by human activities except for occasional visits by bushwalkers and cavers. An extensive deposit of highly karstic and cavernous limestone of Ordovician age outcrops over about 10 km^2^ between 0–300 meters above sea level (asl) on the western and southwestern flanks of Precipitous Bluff (1120 m asl) (Hughes 1957; Dixon and Sharples 1986) (Figure 2). The study area lies within the per-humid precipitation effectiveness province of Gentilli (1972). The nearest meteorological station is at Hastings located 25 km east-northeast, where mean annual rainfall is 1,416 mm. The cool, moist climate supports a dense vegetation cover of wet sclerophyll and cool temperate rainforest (Reid et al. 1999) (Figure 3).

**Figure 4.**
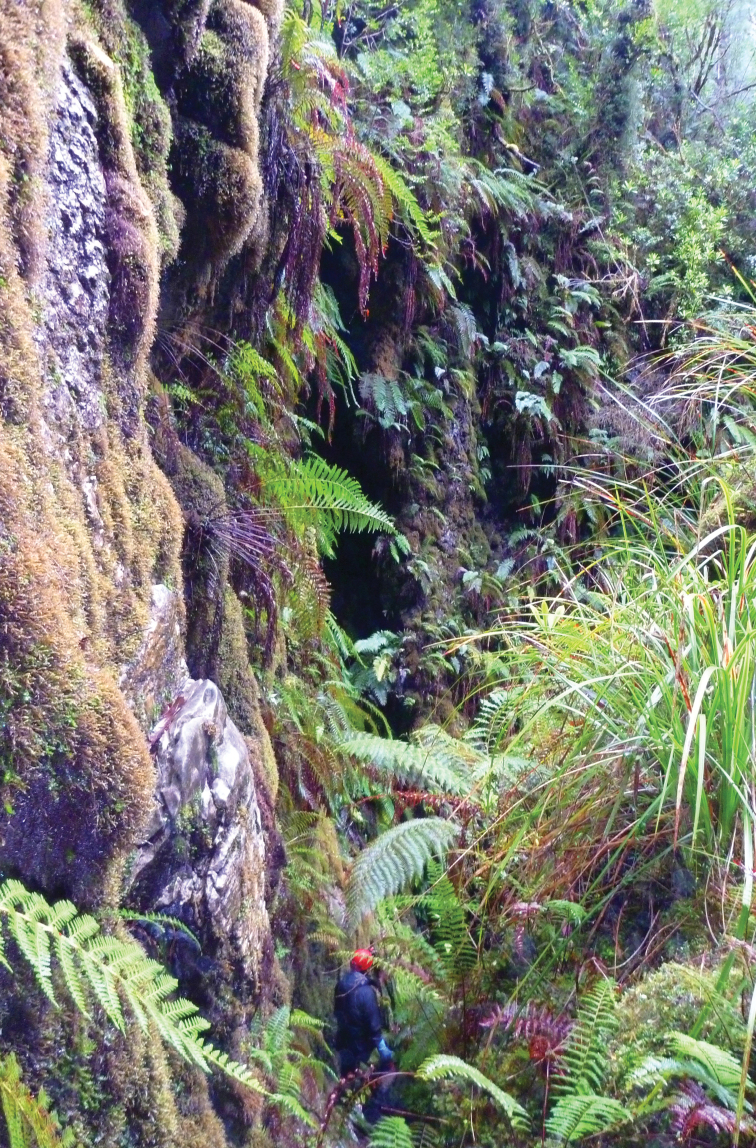
Typical karst terrain and cool temperate rainforest vegetation in the study area.

**Figure 5.**
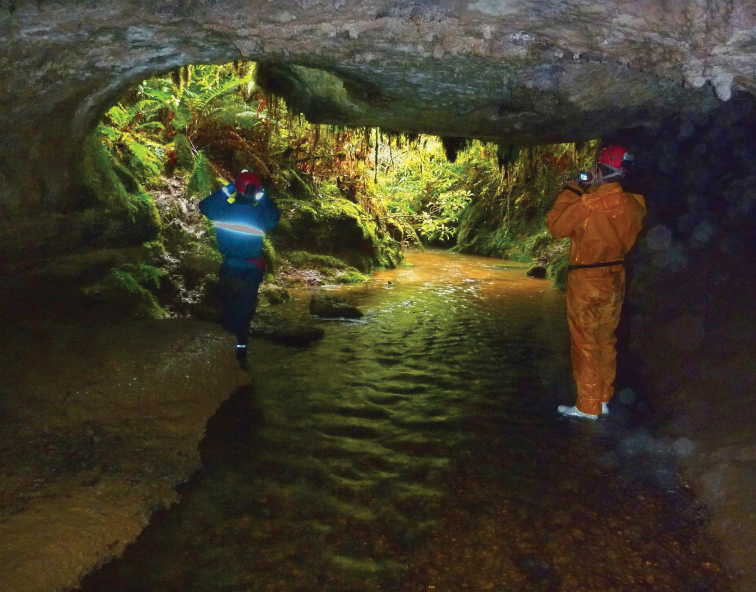
Damper Cave entrance and stream resurgence. Collection sites were located approximately 20 to 80 m inside the cave entrance, see next figures.

The caves have developed by allogenic recharge from streams descending from the upper slopes of the mountain which sink underground on contact with the limestone. Autogenic recharge is also received from rainfall directly on the limestone outcrop, which percolates downward via a multitude of vertical shafts and fissures in the limestone (Figure 4). The descending subsurface flow paths coalesce at lower levels to form an integrated dendritic cave drainage network which discharges from resurgence caves situated at base level on the slope-plain juncture. Damper Cave is a major resurgence cave with more than one km of surveyed underground passages (Figure 5). The cave consists of a major conduit carrying a permanent flowing stream with a bedrock-gravel-sandy bed, in addition to numerous small tributary streams and seepages.

The cave fauna at Precipitous Bluff was surveyed as part of a state-wide biodiversity survey of Tasmanian karsts undertaken by Eberhard et al. (1991). This study discovered that the Precipitous Bluff karst supported the richest assemblage of locally endemic and obligate cave-dwelling species in Tasmania (Eberhard 1996). Initially, most of this assemblage comprised terrestrial cave obligate species (troglobites) including described species of Coleoptera (Eberhard and Giachino 2011; Moore 1978), Diplopoda (Mesibov 2005), Opiliones (Hunt 1990; Hunt and Hickman 1993), synotaxid (Forster et al. 1990) and micropholcommatid (Rix and Harvey 2010) spiders, in addition to other undescribed species of Opiliones, Araneae, Pseudoscorpionida, Oniscidea, and Collembola (Eberhard et al. 1991). A significant fauna of obligate aquatic subterranean species (stygobites) in the Precipitous Bluff caves was also indicated by the discovery of Australia's first stygobitic gastropod *Pseudotricula eberhardi* Ponder 1992, followed by the description of an additional seven species of cave dwelling hydrobiid gastropods (Ponder et al. 2005). Besides the hydrobiids, the sampled aquatic macrofauna included mayflies (Ephemeroptera) and crustaceans (Amphipoda and Syncarida), the latter possessing clear stygomorphies (loss of eyes, pigment and elongation of appendages) indicating their status as stygobites. The initial survey by Eberhard et al. (1991) focused on collecting macro-invertebrates, but Ponder et al. (2005) concluded that additional sampling, especially targeted towards aquatic micro- and meiofauna, was likely to further increase the richness of subterranean species known from the Precipitous Bluff karst. This possibility provided the impetus for the current study.

## Field methods

**Figure 6.**
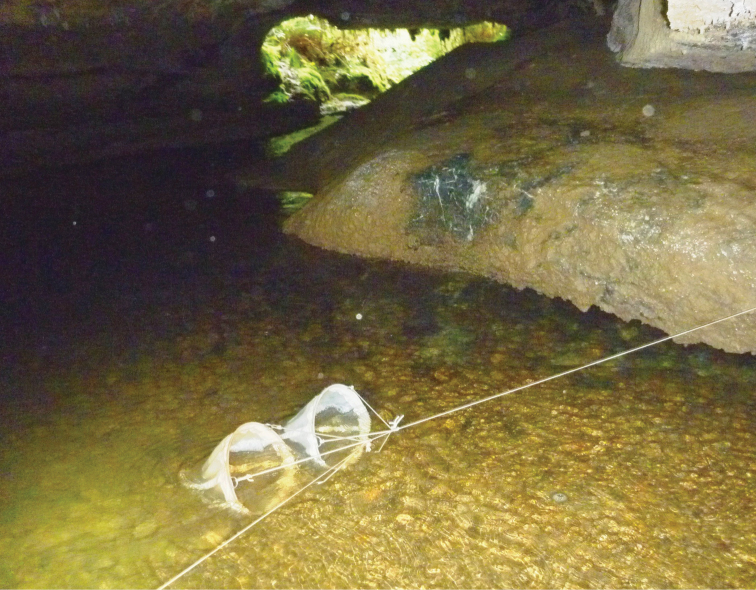
Damper Cave main stream collection Site A with drift nets.

**Figure 7.**
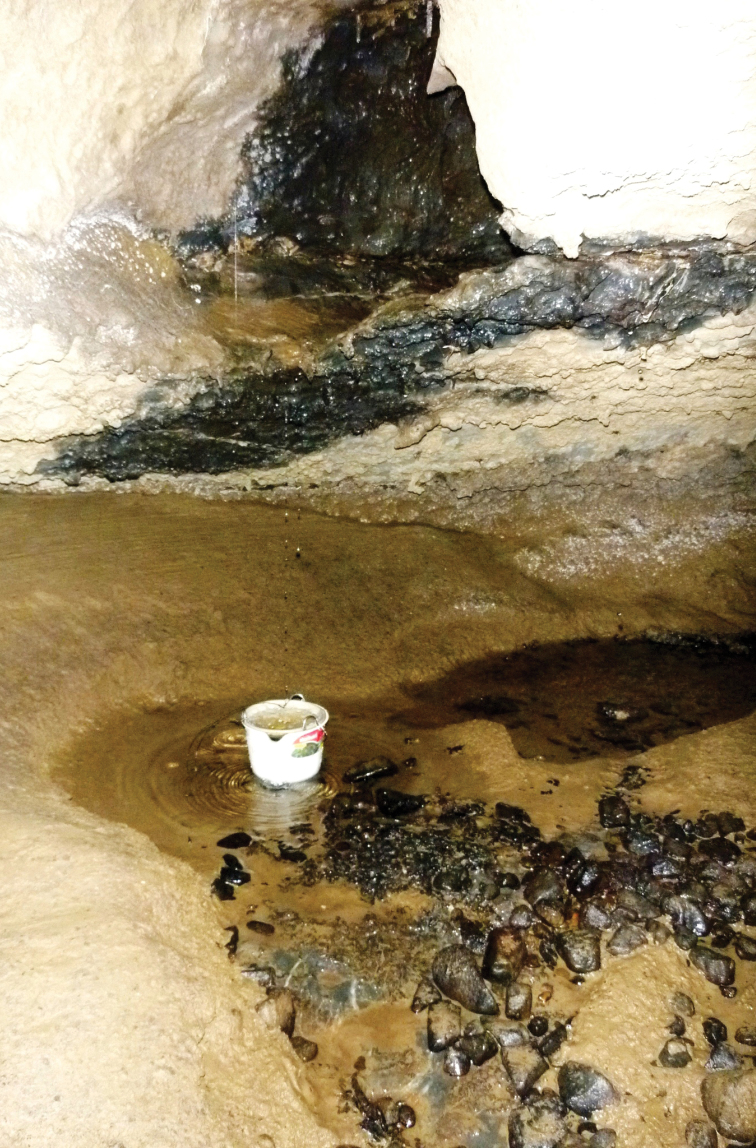
Damper Cave tributary stream collection Site B with net.

Aquatic micro- and meiofauna were sampled from a variety of habitats in Damper Cave in May 2011 using 150 µm mesh plankton nets. Three types of aquatic habitats were sampled: (1) main stream (Site A); (2) small tributary stream (Site B); and (3) seepage water from dripping stalactites (Sites, C, D, E). To sample the main stream benthos, shallow interstitial and stream drift fauna, two 300 mm diameter plankton nets were installed in the main flow of the stream approximately 30 m upstream from the resurgence entrance (Figure 6). Benthic and shallow interstitial fauna was sampled by ‘kick' sampling which involved walking upstream from the nets for a distance of approximately 50 m while ‘kicking' the gravel-sandy sediments to dislodge fauna which was swept downstream into the nets. A small tributary stream approximately 20 m inside the resurgence entrance was sampled by placing a bucket and net underneath a vertical trickle (Figure 7). Seepage water from dripping stalactites was sampled in a similar manner. The nets were left in the cave for two days and then recovered, where after the net contents were elutriated and preserved in 100% ethanol.

## Taxonomic methods

Specimens were dissected and mounted on microscope glass slides in Faure's medium, which was prepared following the procedure of Stock and von Vaupel Klein (1996). The dissected appendages were then covered with a coverslip and observed under the Leica L2 stereomicroscope and Leica DM 2500 compound microscope with N-plan objectives, and the drawings were made using a drawing tube. The photographs of ostracod shells were taken with a Leica M205 C microscope fitted with a Leica DFC420 digital camera, and montaged using the Leica Application Suite V3 software. All material is deposited at the Tasmanian Museum and Art Gallery (TMAG), Hobart.

**Terminology.** In the present paper the terminology for the most posterior appendage on the body, the so-called uropodal ramus, follows Meisch (2007). Terminology of other soft parts follows Broodbakker and Danielopol (1982), Martens (1987), and Meisch (1996).

**Abbreviations used in text and figures.** A1-antennula; A2-antenna; LV-left valve; L5-L7-fifth-seventh limb; Md-mandibula; Mxl-maxillula; RV-right valve; UR-uropodal ramus.

## Taxonomy

### Class Ostracoda Latreille, 1806. Subclass Podocopa Müller, 1894. Order Podocopida Sars, 1866. Superfamily Cypridoidea Baird, 1845. Family Cyprididae Baird, 1845. Subfamily Scottiinae Bronstein, 1947

#### Genus Austromesocypris

Martens, De Deckker and Rossetti, 2004

http://species-id.net/wiki/Austromesocypris

##### Type species.

*Austromesocypris berentsae* Martens, De Deckker and Rossetti, 2004; original designation.

##### Other species.

*Austromesocypris australiensis* (De Deckker, 1983); *Austromesocypris bluffensis* sp. n.; *Austromesocypris tasmaniensis* (De Deckker, 1983)

#### Austromesocypris bluffensis sp. n.

urn:lsid:zoobank.org:act:A2DDB15B-AC3D-433C-9FFC-561AFBFDEAAD

http://species-id.net/wiki/Austromesocypris_bluffensis

**Figure 8.**
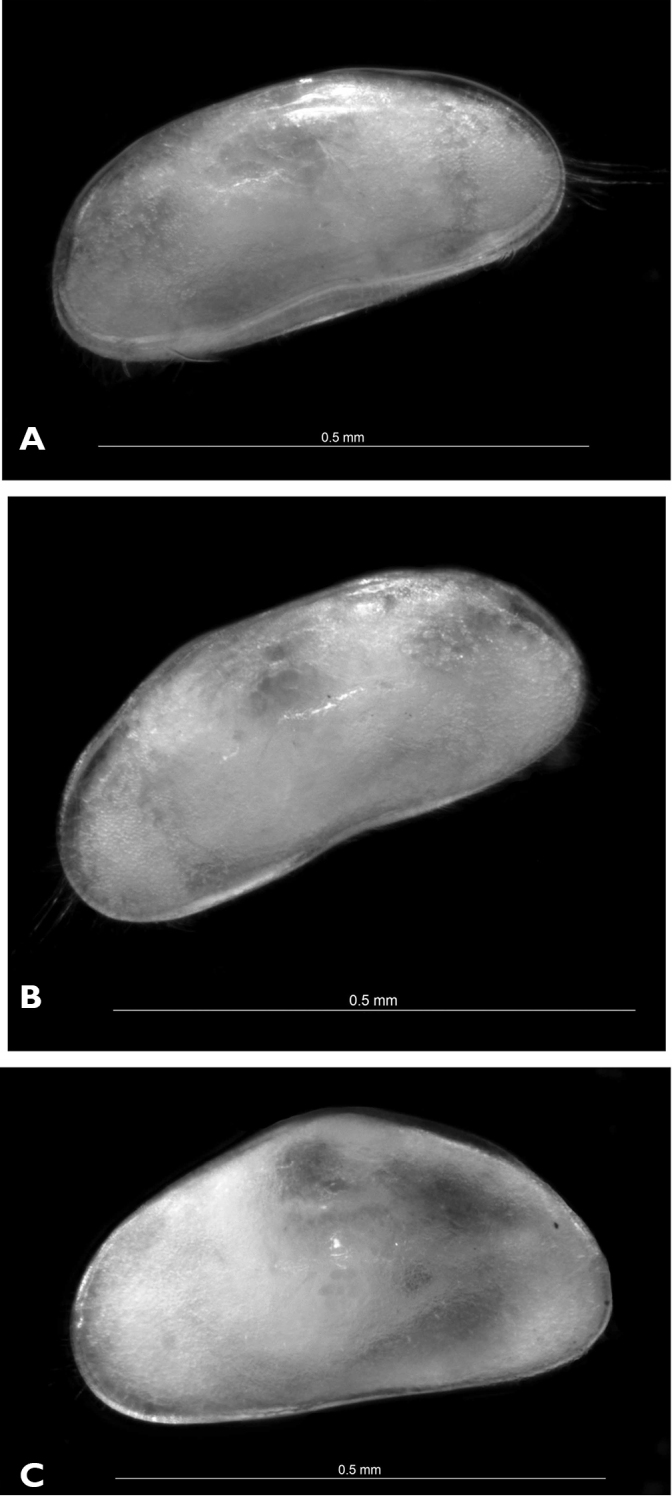
**A, B** *Austromesocypris bluffensis* (Holotype) **C** *Austromesocypris* sp. **A** shell, view from the right side **B, C** shell, view from the left side.

Figures 8A, B 9 [Fig F10] –11

##### Material examined.

Holotype, female (TMAG 6206) Damper Cave, 43°30'S, 146°35'E, Precipitous Bluff, 90 km SW of Hobart, Tasmania, Australia, Site A, main stream, 30 m from entrance, 14 May 2011 (dissected on one slide), collected by R. and S. Eberhard, G. Perina, S. Catomore.

##### Diagnosis.

Ostracods with smooth, transparent shell densely covered with setulae. Dorsal margin straight, anterior and posterior margins almost equally wide. Calcified inner lamella narrow. A1 with fused third and fourth segments. A2 with only two short swimming setae and two t-setae. Md-palp with pappose γ-seta. L5 with one a-seta and one d-seta. L6 with d2-seta, short e-seta, second and third endopodal segments fused, and long terminal claw. UR with row of long, strong setulae along posterior margin; both distal claws strong with strong spines; anterior seta long. Rami almost symmetrical, only one ramus with slightly shorter setulae along posterior margin. Genital field with one clear thumb-like projection.

##### Etymology.

The species is named after its type locality.

##### Description of female.

**Figure 9.**
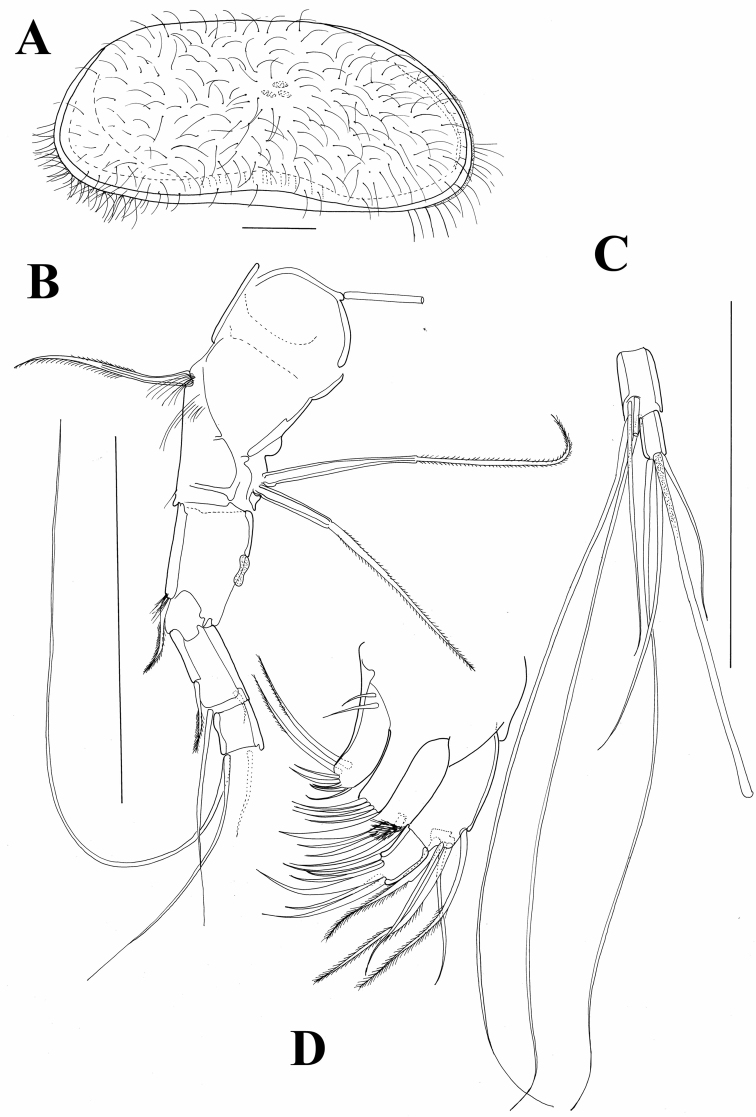
*Austromesocypris bluffensis* (Holotype): **A** shell, lateral view from the right side **B** A1 **C** two distal segments of the A1 **D** Mxl. Scales = 0.1 mm.

*Carapace* (Figs 8A, B; 9A). Rectangular in lateral view; 0.58 mm in length. Greatest height situated around middle length, equalling 0.26 mm, or 45% of length. Valves clearly asymmetrical, RV being shorter than left one. Dorsal margin straight on almost entire length, rounding towards posterior end and inclined with small recess towards anterior end. Both anterior and posterior ends rounded, anterior end more so and slightly wider than posterior one. Ventral margin straight or very slightly concave. Anterior and posterior inner calcified lamellae narrow. Marginal pore canals short, except in ventral region where enlarged. Surface densely covered with relatively short hair-like setae.

*A1* (Fig. 9B, C). First segment hirsute, anteriorly with only one pappose seta, reaching middle length of following segment; posteriorly with two, almost equally long, pappose setae originating close to each other and near distal margin of segment. Wouters organ not observed. Following segment with very short Rome organ posteriorly and one pappose seta anteriorly, reaching middle of following segment. Above this seta cluster of setulae present. Third segment compound, representing fused segments three and four; point of their fusion clearly marked by one short pappose seta, which almost reaches distal margin of following free segment. Distal margin of fused segment with one posterior, short and smooth seta and one long, also smooth seta anteriorly. This seta as long as length of all segments combined. Segment following fused segments (fifth segment) very short with two long and smooth setae anteriorly and one shorter seta posteriorly. Penultimate segment with total of four anterior setae, all situated very close to each other, three being longer than length of all segments combined and one equalling 1/3 of their length. Terminal segment with one long and two shorter setae, this segment with long aesthetasc y1 being eight times longer than terminal segment. Length ratios of last four segments equalling 1.8 : 1 : 1.5 : 1.

**Figure 10. F10:**
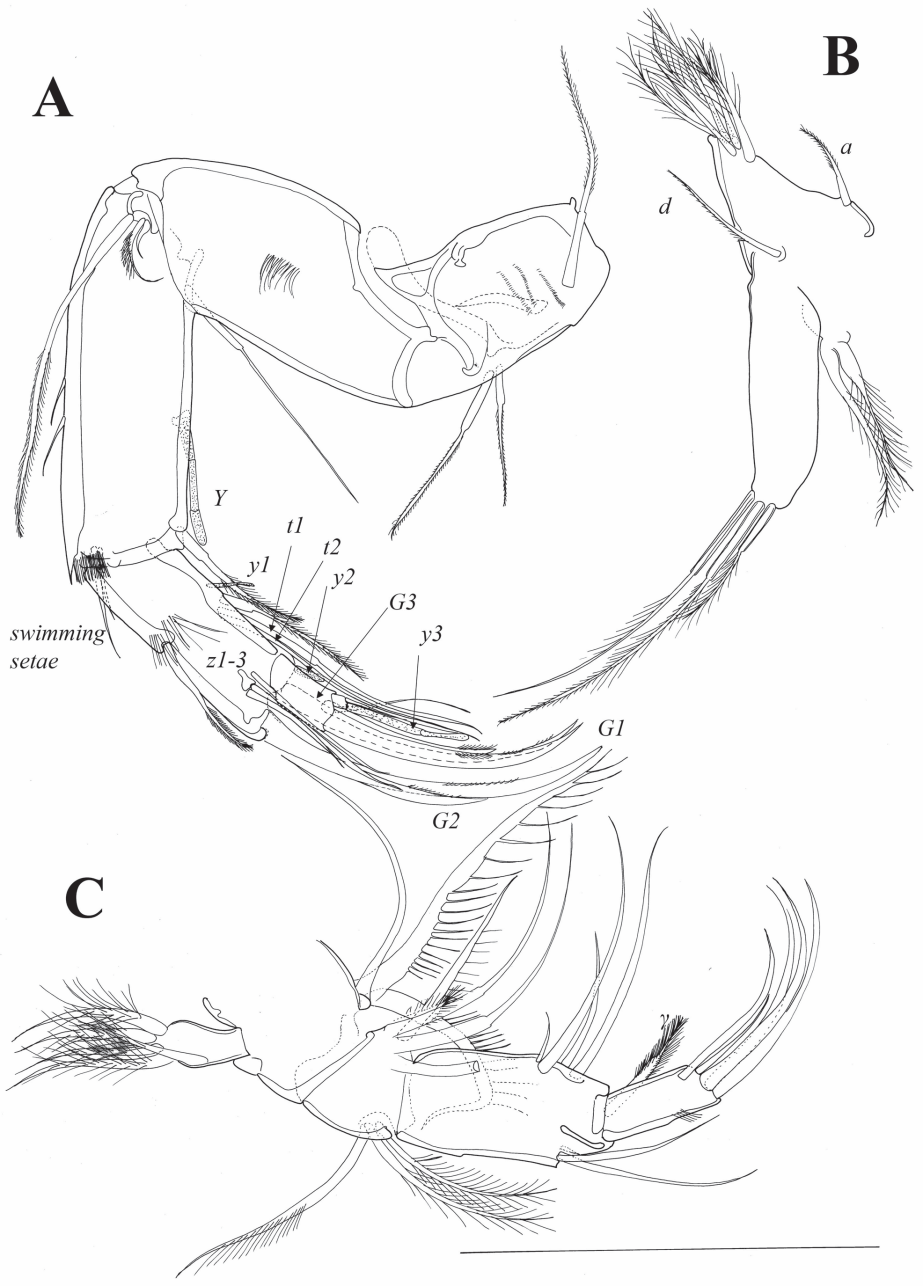
*Austromesocypris bluffensis* (Holotype): **A** A2 **B** L5 **C** Md-palp. Scales = 0.1 mm.

*A2* (Fig. 10A). Coxa with three pappose, equally long setae: one situated more proximally (externally) and two more distally (internally) on segment. Coxa with three rows of small setulae. Basis laterally with cluster of setulae and one anterior seta, which reaches distal end of first endopodal segment. Exopod representing small plate with three setae: most anterior one distally pappose and reaching distal margin of first endopodal segment; middle one also pappose and much shorter; most posterior one even shorter and also smooth. Endopod 3-segmented. First segment with two strong setulae along anterior margin and row of short setulae antero-distally; aesthetasc Y situated in middle of posterior margin and reaching distal end of segment; postero-distal seta pappose and extends beyond middle of second endopodal segment. Two short swimming setae situated antero-distally on segment and only one reaches 1/3 of second endopodal segment. Second endopodal segment with cluster of setulae mid-laterally; seta on middle of anterior margin distally pappose and reaches distal end of segment. Second endopodal segment posteriorly with two short aesthetascs: y1 situated more proximally and y2 at distal margin. Same segment postero-medially with three t-setae: t1 distally pappose and shorter than t2 and t3. Second endopodal segment with three smooth and equally long z-setae on distal margin, all reaching middle of terminal claws. G2 claw equalling 2/3 of G1 claw. G1 and G3 equally long and only slightly shorter than first endopodal segment. Terminal segment short with GM claw equalling 70% of first endopodal segment; Gm claw being 2/3 of GM. Same segment with one additional thin seta which is as long as Gm claw; aesthetasc y3 of same length and proximally fused with one thin seta.

**Figure 11.**
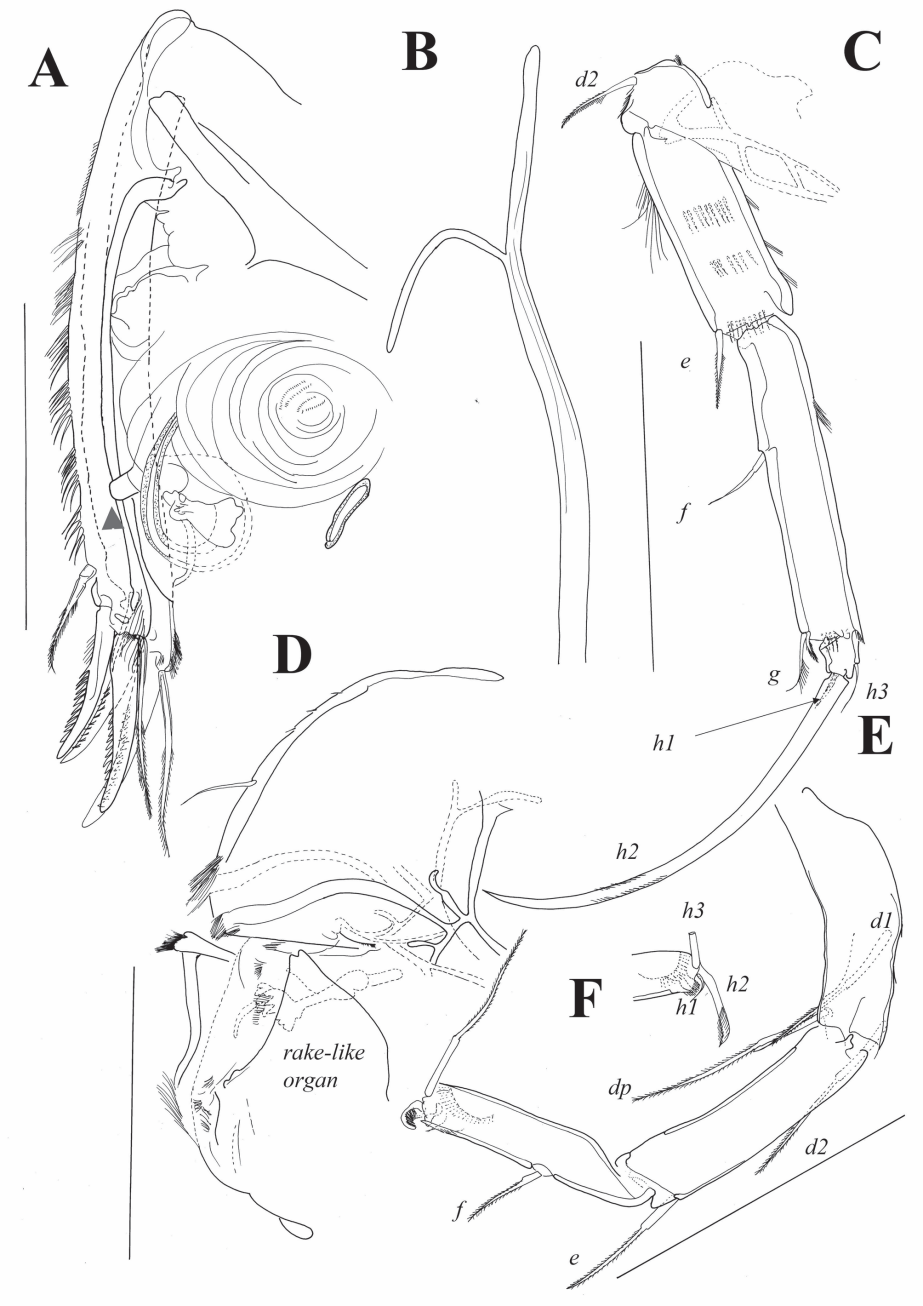
*Austromesocypris bluffensis* (Holotype): **A** UR, arrow indicating the genital process **B** attachment of the UR **C** L6 **D** forehead and upper lip **E** L7 **F** detail of the distal end of L7. Scales = 0.1 mm.

*Forehead and lips* (Fig. 11D). Hirsute with numerous sclerotized rods and rake like organ carrying about six blunt teeth.

*Md* (Fig. 10C). Exopod short carrying five plumose, vibratory setae. Palp 4-segmented. First segment posteriorly with three setae, one smooth and other two with one row of long and strong setulae; one of these setae bent. Alpha seta, usually present on this segment, not observed. Second segment anteriorly with three equally long pappose setae not reaching distal margin of following segment; posteriorly with three long, smooth setae, one shorter seta with setulae along one margin, and one short and plumed seta (β-seta). Penultimate segment anteriorly with one very short seta and two long and smooth setae each exceeding distal end of terminal segment; posteriorly with total of four setae: two long and two shorter, one of which half as long as long setae, other ¼ length of these setae; γ-seta plumed, exceeding distal end of terminal segment. Terminal segment elongated (2.5 times longer than wide) with four distally curved claws and one seta half as long as claws.

*Mxl* (Figure 9D). Palp 2-segmented. First segment with three pappose and two smooth setae, all situated close to outer margin. Terminal segment 1.8 times longer than wide, with two claws (one fused with segment) and four setae. First endite distally with two long, pappose setae and five short claws; proximally with two short and smooth setae. Second endite with four short claws. Third endite with four claws (one fused with segment) and cluster of short setulae on anterior margin.

*L5* (Fig. 10B). Exopod with two plumose vibratory setae. Endopod with three distal pappose setae, two being twice as long as third. Protopod with one pappose a-seta, one pappose d-seta and six distal pappose setae.

*L6* (Fig. 11C). Basal segment (basis) with short pappose d2-seta and marginal rows of setulae. First endopodal segment with long anterior setulae, and three medial rows of shorter setulae; same segment antero-distally with short and pappose e-seta. Following segment compound, representing fused second and third segments, with f-seta near middle of anterior margin and distally with two setae (one being g-seta) and row of marginal setulae. Terminal segment with two thin setae (h1 and h3) and one strong claw (h2); latter gently serrated and 0.72 times as long as endopodal segments combined.

*L7* (Fig. 11E, F). Composed of three segments plus terminal pincer. First segment with three long, pappose setae (d1, d2 and dp). Second segment with pappose e-seta reaching half length of following segment. Third segment compound, representing fused endopodal segments two and three, with f-seta near middle of anterior margin; g-seta absent. Terminal segment reduced and transformed into pincer organ. Seta h1 very thin and curved, h2-seta claw-like and distally pappose, seta h3 normal and almost as long as penultimate segment.

*UR* (Fig. 11A). Posterior margin with groups of long and strong setulae; posterior seta situated close to distal margin, being pappose and more than half as long as posterior claw. Posterior claw only slightly shorter than anterior one; both claws strongly serrated. Anterior seta distally pappose and as long as anterior claw. Rows of setulae on posterior margin of one ramus slightly weaker. Length ratios between anterior margin of ramus and anterior and posterior claws equalling 2.9 : 1.1 : 1.

*Attachment of UR* (Fig. 11B). Distally bifurcate, with no additional ventral or dorsal branches.

*Genital field* (Figure 11A) with clear thumb-like projection (indicated by arrow on Figure 11A).

##### Males.

Not known.

##### Remarks.

*Austromesocypris bluffensis* sp. n. stands apart from all other species of the genus in having a completely flat dorsal margin of the carapace and almost symmetrical UR. In addition, it differs from the New South Wales species, *Austromesocypris berentsae* Martens, De Deckker and Rossetti, 2004, in having only one segment on the A1 fused. *Austromesocypris berentsae* is the only species in the genus that has two segments fused (3+4, 5+6). *Austromesocypris bluffensis* differs from *Austromesocypris australiensis* (De Deckker, 1983) by having structurally similar posterior setae on both UR. Only *Austromesocypris australiensis*, among all species of the genus, has one seta transformed into a strong claw, the other being seta-like. The one previously described species from Tasmania, *Austromesocypris tasmaniensis* (De Deckker, 1983), has much stronger spines along the posterior margin of the UR. The UR of the new species is the most similar to *Austromesocypris berentsae*, but the former species has shorter spine-like setae along the posterior margin of the ramus. It appears that the gamma-seta on the Md-palp in *Austromesocypris berentsae* is not pappose (see Martens et al. 2004, Fig. 3), while it is in the other three species. There are other differences in the chaetotaxy of the Md-palp between the new species and the other three, namely *Austromesocypris bluffensis* has four setae postero-distally on the penultimate segment, *Austromesocypris berentsae* has two, while the two species described by De Deckker (1983) have three. Moreover, there are only two setae antero-distally on the same segment in *Austromesocypris bluffensis*, while all other species have three. It is interesting to note that the S2 seta on the first segment of the Md-palp is turned upwards only in *Austromesocypris bluffensis*. We are uncertain if this character has any taxonomic significance, or whether this seta was twisted during slide preparation. However representatives of many Cyprididae subfamilies (Cypridopsinae, Cyprinotinae, Herpetocypridinae) and some Candoninae and Cyclocypridinae have the seta S2 similar to that of *Austromesocypris bluffensis* (I. Karanovic personal observation).

#### Austromesocypris sp.

Figures 8C 12

##### Material examined.

One juvenile female (A-7 or A-8) (TMAG 6207), Damper Cave, Precipitous Bluff, 43°30'S, 146°35'E, 90 km SW of Hobart, Tasmania, Australia, Tributary stream 20 m inside entrance, 14 May 2011 (dissected on one slide), collected by R. and S. Eberhard, G. Perina, S. Catomore

##### Descriptive notes.

**Figure 12.**
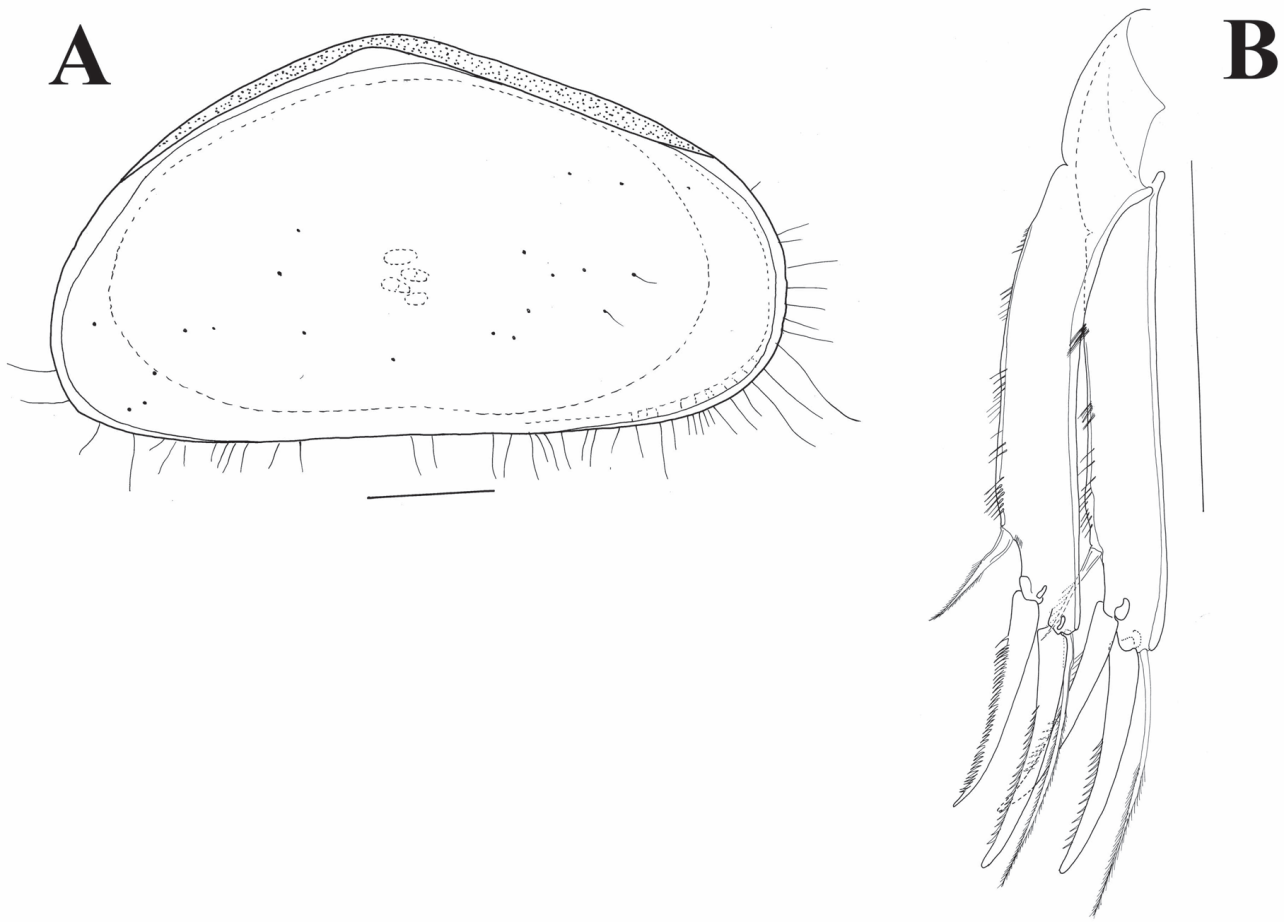
*Austromesocypris* sp.: **A** shell, external view from the right side **B** UR. Scales = 0.1 mm.

Triangular shell (Figs 8C, 12A) with left valve overlapping right one. Anterior and posterior margins rounded with long marginal hair-like setae. Surface not highly hirsute. Length 0.58 mm. UR (Fig. 12B) with anterior margin sparsely covered with strong setulae (same on both rami). Posterior seta pappose and only 1/3 length of posterior claws. Both claws strongly serrated; posterior one only slightly shorter. Anterior seta pappose and longer than anterior claw.

##### Remarks.

This species is assigned to the genus *Austromesocypris* Martens, De Deckker & Rossetti, 2004 based on the appearance of the UR. The undescribed female was probably a late juvenile (A-7 or A-8), as indicated by the well-developed appendages (including the 6-segmented A1). However, the setae on the L6 and L7 had a swollen base and the oviducts, as well as the entire genital field, were undeveloped. Until now, no Scottiinae were known with such a triangular and asymmetrical carapace shape. An unnamed fossil species, *Mesocypris* sp., from the Pulbeena Swamp in Tasmania (De Deckker 1982) also has a highly arched carapace, but the posterior margin in this species is broader and the LV is not higher than the RV.

##### Associated fauna.

Other taxa collected in the Damper Cave samples included: a) Copepoda (*Diacyclops cryonastes* Morton, 1985), Amphipoda (Paramelitidae), Isopoda (Styloniscidae), Oligochaeta (Phreodrilidae), Gastropoda (Hydrobiidae), Nematoda, Turbellaria (Tricladida), Diptera, Plecoptera, and Ephemeroptera from the main stream Site; and b) Isopoda (Janiridae: *Heterias* sp.) from the stalactite drip Site E.

## Discussion

### Ecology

No Ostracoda have previously been described from Tasmanian caves or other groundwater environments. Illife (1988) reported a collection of ostracods from Honeycomb Cave at Mole Creek in northern Tasmania; however these remain unidentified. As already noted, biospeleological collections to date have targeted macro-invertebrates while aquatic micro- and meio-fauna have been poorly investigated (Ponder et al. 2005). The absence of Candoninae in Tasmanian caves may be an artefact of poor sampling effort, but it is also possible that this group is substituted here by *Austromesocypris* Martens, De Deckker & Rossetti, 2004 species that have occupied a new habitat through the lack of competition with Candoninae, the dominant subterranean ostracod group elsewhere in the world (Karanovic 2007). Despite a substantial sampling effort during this study, only one complete specimen of each species has been collected. Subterranean ostracods are often rare in subterranean samples (Karanovic 2007, Karanovic and McKay 2010).

Discovery of cave dwelling ostracods described in this paper is of interest for two reasons. Firstly, members of Cyprididae are rarely found in subterranean waters. Secondly, the genus *Austromesocypris* is otherwise known to comprise entirely “terrestrial and semi-terrestrial” species. The rare finds of Cyprididae in subterranean waters are mostly records of morphologically unmodified taxa which occur as facultative or incidental inhabitants of groundwaters. The only documented case of an obligate association of cyprids with groundwaters involves the genus *Pseudocypridopsis* Karanovic, 1999, with two described stygobitic species from the Balkan Peninsula (Karanovic 1999, 2000). In Western Australia, where a great deal of sampling in wells and water bores has revealed a globally significant richness of stygobitic Crustacea (Eberhard et al. 2005, 2009), no truly stygobitic Cyprididae occur, although unmodified epigean species are not infrequently collected especially in wells which are open to the surface and therefore easily colonized by epigean species.

The collection of a whole specimen of *Austromesocypris bluffensis* sp. n. in the main stream implies that this animal could have been living in the benthos or interstitial of the main stream, but also does not preclude an origin from a tributary stream or seepage waters, of which the latter two ultimately discharge into the main stream. The collection of valves in seepage water from dripping stalactites and a complete specimen of *Austromesocypris* sp. in the small tributary stream confirms the occurrence of *Austromesocypris* in these habitats also. Notwithstanding they might still have originated from the surface which lies only a few tens of meters above Damper Cave at the point where the collections were made. Certainly the cool temperate rainforest represents a near-permanently moist habitat for hygrophilous invertebrates including, potentially, terrestrial/semi-terrestrial ostracods. The highly permeable karst at Precipitous Bluff ensures a close eco-hydrological connection between epigean and hypogean environments, confirmed by the occurrence of other normally epigean-terrestrial invertebrates such as charopid gastropods accidentally washed into the caves by percolating seepage waters (S. Eberhard personal observation).

### Adaptive morphology

Some adaptive morphologies, typically comprising numerous reductions, displayed in semi-terrestrial epigean ostracods are similar to those seen in aquatic subterranean ostracods as already pointed out by several authors (Danielopol 1978, Danielopol and Betsch 1980, Pinto et al. 2005, 2008). Danielopol and Betsch (1980) also point out the close phylogenetic relationships between the semi-terrestrial Candoninae from Madagascar and their interstitial aquatic relatives. This may additionally support the view of Karanovic (2007) that the subterranean Candoninae genus *Nannocandona* Eckman, 1914 should belong to Terrestricandonini instead of Candonini because of the many morphological characters shared with members of the former tribe. It is also worth noting that the subfamily Timiriaseviinaenot only contains the only freshwater semi-terrestrial cytheroid ostracod *Intrepidocythere* Pinto, Rocha and Martens, 2008 but also is the only cytheroid subfamily with true stygobiont species such as those in the genera *Kovalevskiella* Klein, 1963 and *Gomphodella* De Deckker, 1981.

The unusual shape of the two new Tasmanian species, one with trapezoidal shape, the other with triangular shape, suggests that these might also be true stygobites as such modification is very common among subterranean Candoninae (Danielopol 1978, Karanovic 2007). In addition to this potentially convergent stygomorphic character, typical for Ostracoda, the *Austromesocypris* from Precipitous Bluff also exhibit classic convergent regressive stygomorphic characters seen in subterranean Crustacea, including depigmentation, loss of eyes and reduction in body size. On the other hand, these Tasmanian specimens do not exhibit any other obvious stygomorphic characters such as elongation of appendages as seen in Candoninae and most other stygomorphic Crustacea. It should be noted that the dominantly terrestrial/semi-terrestrial group Scottiinae typically exhibit reduction in body size and size of segments, a gracile appearance, and anopthalmia.

### Systematics

*Austromesocypris* is according to Martens et al. (2004) most closely related to *Mesocypris* Daday, 1910 and they share a reduced number of free segments on the A1, fused second and third endopodal segments on the L6, a short h3 seta on the terminal segment of the same appendage, and asymmetrical caudal rami. *Mesocypris* is a paraphyletic taxon according to the cladistic tree obtained by Martens et al. (2004), and indeed many species deviate from the characters that should define the tribe Mesocypridini to which the two genera belong according to Martens et al. (2004). For example, at least two species have the second and third endopodal segments partly separated on the L6: *Mytilocypris terrestris* Harding, 1953 and *Mytilocypris pauliani* Danielopol & Betsch, 1980. The h3 seta is longer in all *Mesocypris* species than in *Austromesocypris* and its length is somewhere in between for the latter genus and *Scottia* Brady & Norman, 1889. Martens et al. (2004) stated in their revised diagnosis that in tribe Mesocypridini h3 is short, while in Scottiini it is claw-like. One of the Madagascar species, *Mytilocypris pauliani* Danielopol & Betsch, 1980 actually has a claw-like h3 seta. Another doubtful character of the tribe Mesocypridini is the morphology of the UR. While the posterior margins of the right and left ramus are indeed asymmetrical (one hirsute, other with teeth or spines) in all previously known species, only *Austromesocypris australiensis* has one posterior seta transformed into a strong claw and the other seta-like. In all other Mesocypridini species this difference is much less obvious, especially in other species of the genus *Austromesocypris*.

All species of *Austromesocypris* have only two swimming setae on the A2, while in *Mesocypris* the number varies from six to two. There are, however, two characters which indicate the two genera are more closely related to each other than either is to the genus *Scottia*. Namely, the terminal segment on the Md-palp is much more elongate and the number of setae on the first segment of the Mxl-palp is reduced in *Mesocypris* and *Austromesocypris* in comparison to *Scottia*. According to Chapman's (1961) illustration of the Md of *Scottia audax* (Chapman, 1961)and Chapman's (1963) drawings of the Mxl of *Scottia insularis* Chapman, 1963,the two New Zealand species appear to be more closely related to members of Mesocypridini. *Scottia audax* was originally described in the genus *Mesocypris*, but De Deckker (1980) transferred it to the genus *Scottia* because of the morphology of the L6, namely the presence of the long, claw-like h3 seta, and at the same time also suggested that *Scottia insularis* Chapman, 1963 might belong to *Mesocypris* due to the short h3 seta. Both New Zealand species were retained in the genus *Scottia* by Martens et al. (2004), but those authors pointed out the unusual distribution of the genus, with two species, *Scottia pseudobrowniana* Kempf, 1971 widely distributed in the Holarctic (Meisch 2000) and *Scottia birigida* Smith, Matzke-Karasz, Kamiya & Ikeda, 2002 endemic to Japan (Smith et al. 2002).

The position of the two New Zealand species is not certain at the moment, but they definitely should be excluded from the genus *Scottia*. While they share many characters with *Mesocypris*, most of which are variable, they also have a reduced number of segments on the A1 like *Austromesocypris*. Fusion of A1 segments can also be partial, as noted by Matzke-Karasz (1995) for *Scottia audax*. Namely, borders between segments 3+4 and 5+6 are incompletely formed and only visible on the interior side, a fact noticed by Martens et al. (2004) as well. Another character that may support a closer relationship of *Scottia audax* to Mesocypridini is the reduced number of rays on the exopod of L5. Namely, both Palearctic *Scottia* species have six rays, while the New Zealand species has only four (see drawings of Matzke-Karasz 1995). The number is further reduced to two or three setae in *Mesocypris* and *Austromesocypris*. This character needs to be taken with caution, because the exopod of L5 may be easily damaged during slide preparation and the setae easily fall off.

### Misidentified species

In our opinion Australian records of *Scottia audax* (Chapman, 1961) by De Deckker (1980) are incorrect, that material representing a different, as yet undescribed species. This is even more apparent from the drawings of *Scottia audax* provided by Matzke-Karasz (1995) who studied De Deckker's (1980) material. The most significant difference between the Australian species and *Scottia audax* is the morphology of the UR. The posterior seta is longer, stronger and more closely situated to the distal end of the ramus in the Australian species, but the claws are stronger and the posterior margin of the ramus is covered with small teeth (or at least thick spines) in *Scottia audax*. Another finer detail is the appearance of the exopodal setae of the A2, which in the Australian species has one long and two short, subequal setae whereas in *Scottia audax* the two shorter setae are unequal in length, with one being at least twice as long as the other (as in *Austromesocypris bluffensis*). The chaetotaxy of the Md-palp is also somewhat different, as all the dorsal setae in *Scottia audax* are longer than those of the Australian species. It is interesting that *Scottia audax* and the Australian species have the S2 seta turned the same way as in *Austromesocypris bluffensis* sp. n. and that all three species also have four setae postero-distally on the penultimate segment.

In our opinion De Deckker's (1983) report of *Austromesocypris australiensis* also represents two species which differ most significantly in the appearance of the hemipenis, prehensile palps and the number of the rosettes on the Zenker organ. One was found in far north Queensland, and the other in New South Wales and Victoria, but both were identified as *Austromesocypris australiensis* by De Deckker (1983). Previous collections of *Austromesocypris tasmaniensis* may also contain two species, as De Deckker (1983) observed some differences in the length of the shell and morphology of the UR between specimens taken from two localities. These samples need to be studied in more detail to draw an accurate conclusion.

Misidentified Scottiinae are not confined to Australian records. Reports of the “widely distributed” *Scottia pseudobrowniana* from Tennessee (Cole 1966) and Nevada (Külköylüoglu and Vinyard 2000) are also probably incorrect. The specimens from Tennessee (east) resemble *Scottia pseudobrowniana* more than those from Nevada (west). While the Nevada material has an almost straight posterior margin of the shell, the one from Tennessee has a rounded posterior margin, similar to that of *Scottia pseudobrowniana*, but with both ends being narrower than in the latter species. Specimens of the North American *Scottia pseudobrowniana* have shorter swimming setae on the A2 than the European *Scottia pseudobrowniana*. Indeed the setae in the Nevada material are so short that they only slightly overpass the first endopodal segment. Specimens of *Scottia pseudobrowniana* from the three separate geographic locations differ mostly in the morphology of the hemipenis. Although neither Cole (1966) nor Külköylüoglu and Vinyard (2000) labelled or drew this organ in detail, the outline they provide indicates enough differences to claim that three separate species are represented. *Scottia pseudobrowniana* has all segments on the A1 free, while Külköylüoglu and Vinyard (2000) illustrated a 6-segmented A1. If this is correct, it would represent an outstanding character. It must be noted, however, that the illustrations of Külköylüoglu and Vinyard (2000) show limited detail and may not correctly show the relevant characters.

*Scottia pseudobrowniana* from Russia, as illustrated by Bronstein (1947), is very similar to the figures of the Nevada species of Külköylüoglu and Vinyard (2000), but they differ in the length of the swimming setae (longer in the Russian species). Bronstein's (1947) illustration of the hemipenis is also rather similar to the figures given by Külköylüoglu and Vinyard (2000). Smith et al. (2002) also noted that *Scottia pseudobrowniana* reported from Russia and Nevada are quite different from the European populations, and suggested that the respective material needs to be re-identified. We conclude that the Russian and both North American records of *Scottia pseudobrowniana* represent new species and that the differences cannot be attributed to variability. This is supported by several reports of the absence of morphological variability among European populations of *Scottia pseudobrowniana* (see Kempf 1971, Danielopol and Vespremenau 1964, Petkovski 1966, Matzke-Karasz 1995, Meisch 2000).

### Key to genera and species of Scottiinae

**Table:** 

1	First segment of the Mxl-palp with seven or eight setae, terminal segment of the Md-palp maximum 1.5 times longer than wide, “e” and “f” setae on L6 long and equal	2
–	First segment of the Mxl-palp with maximum five setae, terminal segment of the Md-palp at least two times longer than wide, “e” seta rarely long, and if so, “f” seta equals half its length	6
2	Swimming setae on the A2 only slightly exceed distal end of the first endopodal segment	[non] *Scottia pseudobrowniana* Kempf, 1971 in Külköylüoglu and Vinyard (2000)
–	Swimming setae on the A2 longer	3
3	Females in lateral view with dorsal margin evenly rounded towards both anterior and posterior ends, so that both ends equally wide and relatively narrow; males with inclined distal margin on the medial lobe of the lateral shield of the hemipenis towards dorsal side, and medial lobe of the lateral shield with a very short dorsal margin	[non] *Scottia pseudobrowniana* Kempf, 1971 in Cole (1966)
–	Females in lateral view with clearly wider posterior margin and the dorsal margin more broadly rounded towards posterior than towards anterior end; males with distal margin of the medial lobe rounded, but almost parallel to the rest of the hemipenis and this lobe with a longer dorsal margin	4
4	Medial lobe of the lateral shield on the hemipenis very narrow, almost finger like	[non] *Scottia pseudobrowniana* Kempf, 1971 in Bronstein (1947)
–	Medial lobe of the lateral shield rectangular- to square-shaped	5
5	Dorsal lobe of the lateral shield on the hemipenis with a prominent projection	*Scottia birigida* Smith et al., 2002
–	No such projection present	*Scottia pseudobrowniana* Kempf, 1971
6	At least one of the UR posterior seta elongated and seta-like, reaching at least half of the posterior claw	7
–	This seta very short, thick and claw-like on both UR	14
7	Penultimate segment on the L6 divided	8
–	Penultimate segment fused	10
8	Seta “h3” on the L6 reaching or exceeding half the length of the terminal claw	9
–	This seta at most reaching 1/3 of the length of the terminal claw	*Scottia insularis* Chapman, 1963
9	Posterior margin of the UR covered with strong spines, and anterior end of the carapace more elongated	*Scottia audax* (Chapman, 1961)
–	Posterior margin of the UR covered with small spine-like setae and carapace in lateral view more tumid	[non] *Scottia audax* (Chapman, 1961) in De Deckker (1980) and Matzke-Karasz (1975)
10	Four segments on the A1 fused, so that A1 is 5-segmented	*Austromesocypris berentsae* Martens, De Deckker & Rossetti, 2004
–	Only two segments on the A1 fused, so that A1 is 6-segmented	11
11	One posterior seta on UR transformed into a thick claw, other more slender and seta-like	*Austromesocypris tasmaniensis* (De Deckker, 1983)
–	Both posterior setae slender	12
12	Shell with straight dorsal margin, shape almost rectangular	*Austromesocypris bluffensis* sp. n.
–	Shell with gently rounded dorsal margin, shape more reniform	13
13	Zenker organ with 10 rosettes of spines, hemipenis slender	[non] *Austromesocypris australiensis* (De Deckker, 1983) in De Deckker (1983, Figure 5A–G)
–	Zenker organ with more than 15 rosettes of spines, hemipenis robust	*Austromesocypris australiensis* (De Deckker, 1983)
14	Seta “h3” on the L6 claw-like	*Mesocypris pauliani* Danielopol & Betsch, 1980
–	Seta “h3” on the L6 seta-like	15
15	Anterior seta on the UR short, not reaching 1/3 the length of the anterior claw	*Mesocypris pubescens* Daday, 1910
–	This seta much longer, exceeding half the length of the anterior claw	16
16	Seta “e” on the L6 long, almost reaching the distal end of the penultimate segment	*Mesocypris terrestris* Harding, 1953
–	Seta “e” on the L6 much shorter, not reaching middle of the penultimate segment	*Mesocypris madagascariensis* Danielopol & Betsch, 1980

## Supplementary Material

XML Treatment for Austromesocypris

XML Treatment for Austromesocypris bluffensis

XML Treatment for Austromesocypris

## Appendix

List of ostracod species considered “terrestrial or semi-terrestrial” or often associated with those habitats

Superfamily Cypridoidea Baird, 1845

Family Cyprididae Baird, 1845

Subfamily Callistocypridinae Schornikov, 1980

Genus *Callistocypris* Schornikov, 1980

*Callistocypris mckenzie* Pinto, Rocha & Martens, 2005: Saõ Paolo State, Brazil (Pinto et al. 2005)

*Callistocypris rossetti* Pinto, Rocha & Martens, 2005: Saõ Paolo State, Brazil (Pinto et al. 2005)

*Callistocypris zlotini* Schornikov, 1980: Solomon Islands (Schornikov 1980)

Subfamily Cypridopsinae Kaufmann, 1900

Genus *Bryocypris* Røen, 1956

*Bryocypris grandipes* Røen, 1956: Cameroon (Martens 1989)

Subfamily Scottinae Bronstein, 1947

Tribe Mesocypridini Puri, 1974

Genus *Austromesocypris* Martens, De Deckker & Rossetti, 2004

*Austromesocypris australiensis* (De Deckker, 1983): Victoria, Australia (De Deckker 1983)

*Austromesocypris bluffensis* sp. nov.: Tasmania (present paper)

*Austromesocypris berentsae* Martens, De Deckker & Rossetti, 2004: New South Wales, Australia (Martens et al. 2004)

*Austromesocypris tasmaniensis* (De Deckker 1983): Tasmania, Australia (De Deckker 1983)

Genus *Mesocypris* Daday, 1910

*Mesocypris madagascariensis* Danielopol & Betsch, 1980: Madagascar (Danielopol and Betsch 1980)

*Mesocypris pauliani* Danielopol & Betsch, 1980: Madagascar (Danielopol and Betsch 1980)

*Mesocypris pubescens* Daday, 1910: Kilimanjaro (Daday 1910), Kenya (Klie 1939)

*Mesocypris terrestris* Harding, 1953: Malawi (Harding 1953)

Tribe Scottini Martens, De Deckker & Rossetti, 2004

Genus *Scottia* Brady & Norman, 1889

*Scottia audax* Chapman, 1961: New Zealand (Chapman 1961), Australia (De Deckker 1980, 1983)

*Scottia birigida* Smith, Matzke-Karasz, Kamiya & Ikeda, 2002: Japan (Smith et al. 2002)

*Scottia insularis* Chapman, 1963: New Zealand (Chapman 1963)

*Scottia pseudobrowniana* Kempf, 1971: Holarctic (Meisch 2000, Cole 1966, Külköylüoglu and Vinyard 2000)

Family Candonidae Kaufmann, 1900

Subfamily Candoninae Kaufmann, 1900

Tribe Terrestricypridini Schornikov 1980

Genus *Caaporacandona* Pinto, Rocha & Martens, 2005

*Caaporacandona iguassuensis* Pinto, Rocha & Martens, 2005: Paraná State, Brazil (Pinto et al. 2005)

*Caaporacandona shornikovi* Pinto, Martens & Rossetti, 2005: Paraná State, Brazil (Pinto et al. 2005)

Genus *Terrestricandona* Danielopol & Betsch, 1980

*Terrestricandona minuta* Danielopol & Betsch, 1980: Madagascar (Danielopol and Betsch 1980)

Genus *Terrestricypris* Schornikov, 1980

*Terrestricypris arborea* Schornikov, 1980: Solomon Islands (Schornikov 1980)

*Terrestricypris wurdigae* Pinto, Rocha & Martens, 2005: Saõ Paolo State, Brazil (Pinto et al. 2005)

Superfamily Cytheroidea Baird, 1850

Family Limnocytheridae Klie, 1938

Subfamily Timiriaseviinae Mandelstam, 1960

Genus *Intrepidocythere* Pinto, Rocha & Martens, 2005

*Intrepidocythere ibipora* Pinto, Rocha & Martens, 2005: Saõ Paolo State, Brazil (Pinto et al. 2005)

Superfamily Terrestricytherioidea Schornikov, 1969

Family Terrestricytheridae Schornikov, 1969

Genus *Terrestricythere* Schornikov, 1969

*Terrestricythere elisabethae* Horne, Smith, Whittaker & Murray, 2004: England (Horne et al. 2004)

*Terrestricythere ivanovae* Schornikov, 1969: Kuril Islands (Schornikov 1969)

*Terrestricythere pratensis* Schornikov, 1980: Vladivostok, Russia (Schornikov 1980)

*Terrestricythere proboscidea* Hiruta, Hiruta & Mawatari, 2007 (Hiruta et al. 2007)

Superfamily Darwinuloidea Brady & Norman, 1889

Family Darwinulidae Brady & Norman, 1889

Genus Penthesilenula Rossetti & Martens, 1998

*Penthesilenula aotearoa* (Rossetti, Eagar & Martens, 1998): Saõ Paolo State, Brazil (Pinto et al. 2004, 2005)

*Penthesilenula brasiliensis* (Pinto & Kotzian, 1961): Saõ Paolo State, Brazil (Pinto et al. 2004, 2005)

*Penthesilenula reidae* Pinto, Rocha & Martens, 2004: Saõ Paolo State, Brazil (Pinto et al. 2004)

Genus *Vestalenula* Rossetti & Martens, 1998

*Vestalenula botocuda* Pinto, Rocha & Martens, 2003: Saõ Paolo State, Brazil (Pinto et al. 2003)

*Vestalenula irajai* Pinto, Rocha & Martens, 2003: Saõ Paolo State, Brazil (Pinto et al. 2005)
